# Effect of taurine supplementation on hyperhomocysteinemia and markers of oxidative stress in high fructose diet induced insulin resistance

**DOI:** 10.1186/1758-5996-2-46

**Published:** 2010-06-30

**Authors:** Hala O El Mesallamy, Ebtehal El-Demerdash, Lamiaa N Hammad, Hekmat M El Magdoub

**Affiliations:** 1 Department of Biochemistry, Faculty of Pharmacy, Ain Shams University, Cairo, Egypt; 2 Department of Pharmacology & Toxicology, Faculty of Pharmacy, Ain Shams University, Cairo, Egypt; 3 Department of Biochemistry, Faculty of Pharmacy, Misr International University, Cairo, Egypt

## Abstract

**Background:**

High intake of dietary fructose is accused of being responsible for the development of the insulin resistance (IR) syndrome. Concern has arisen because of the realization that fructose, at elevated concentrations, can promote metabolic changes that are potentially deleterious. Among these changes is IR which manifests as a decreased biological response to normal levels of plasma insulin.

**Methods:**

Oral glucose tolerance tests (OGTT) were carried out, homeostasis model assessment of insulin resistance (HOMA) was calculated, homocysteine (Hcy), lipid concentrations and markers of oxidative stress were measured in male *Wistar *rats weighing 170-190 g. The rats were divided into four groups, kept on either control diet or high fructose diet (HFD), and simultaneously supplemented with 300 mg/kg/day taurine via intra-peritoneal (i.p.) route for 35 days.

**Results:**

Fructose-fed rats showed significantly impaired glucose tolerance, impaired insulin sensitivity, hypertriglyceridemia, hypercholesterolemia, hyperhomocysteinemia (HHcy), lower total antioxidant capacity (TAC), lower paraoxonase (PON) activity, and higher nitric oxide metabolites (NOx) concentration, when compared to rats fed on control diet. Supplementing the fructose-fed rats with taurine has ameliorated the rise in HOMA by 56%, triglycerides (TGs) by 22.5%, total cholesterol (T-Chol) by 11%, and low density lipoprotein cholesterol (LDL-C) by 21.4%. Taurine also abolished any significant difference of TAC, PON activity and NOx concentration among treated and control groups. TAC positively correlated with PON in both rats fed on the HFD and those received taurine in addition to the HFD. Fructose-fed rats showed 34.7% increase in Hcy level. Taurine administration failed to prevent the observed HHcy in the current dosage and duration.

**Conclusion:**

Our results indicate that HFD could induce IR which could further result in metabolic syndrome (MS), and that taurine has a protective role against the metabolic abnormalities induced by this diet model except for HHcy.

## Introduction

Soft drink consumption has recently been linked with increased risk for weight gain, type 2 diabetes mellitus (T2DM) and other features of the MS [1]. Many studies suggest that the mechanism by which sugar or high fructose corn syrup may induce MS is related to the fructose content [2].

Concern has arisen because of the realization that fructose, at elevated concentrations, can promote metabolic changes that are potentially deleterious. Among these changes is IR which manifests as a decreased biological response to normal levels of plasma insulin and is indicated by impaired glucose tolerance, hyperglycemia and hyperinsulinemia [3].

Metabolic dyslipidemia is the most common complication of IR and T2DM. It is believed to be exacerbated by obesity, as well as numerous detrimental environmental factors such as a high fat diet and sedentary lifestyle. The dyslipidemia accompanying IR is characterized by distinct changes that significantly contribute to increased risk of cardiovascular diseases (CVD) [4].

In addition, elevated levels of glucose, insulin, advanced glycation endproducts (AGEs), TGs and free fatty acids (FFAs) in patients with IR and MS are reported to produce reactive oxygen species (ROS). ROS can in turn elevate tension of redox stress, and cause damage of pancreatic islets [5]. It can also shift the nitric oxide synthase (NOS) reaction towards the production of peroxynitrite (ONOO^-^) rather than nitric oxide (NO), which might contribute to the development of CVD [6].

Hyperhomocysteinemia is accused of being responsible for elevating oxidative stress as a result of formation of Hcy thiolactone, which leads to impairment of insulin signaling and causes IR [7]. Case-control studies have shown a significant association between plasma Hcy and insulin levels in human and animal models [8,9]. However, it is still not clear whether HHcy induces IR or it is actually hyperinsulinemia that causes elevated plasma Hcy levels. The direction of the causality in this association is still controversial. It was previously reported that high concentrations of serum insulin are associated with an increased risk of developing HHcy [10]. However, other *in vitro *studies suggested that Hcy could exert deleterious effects on insulin secretion, resulting in IR [11].

Amino acids have been recognized as important signaling mediators in different cellular functions. Taurine (2-amino ethane sulphonic acid) is a conditionally essential amino acid that is involved in many important biological functions [12]. Taurine reduces the rate of apoptosis in pancreatic islets and acts on DNA synthesis, preventing abnormal development of the endocrine pancreas [13]. In fetal rat islets, taurine increases glucose-stimulated insulin secretion and enhances the action of some secretagogues, such as leucine or arginine [14]. Furthermore, there is evidence indicating that taurine has hypoglycemic properties due to the potentiation of the effects of insulin [15]. Finally, taurine antioxidant properties protect pancreatic beta-cells against oxidative stress-induced decrease in function observed in some pathophysiological conditions [16]. These findings indicate that taurine is involved in distinct central and peripheral processes necessary for the control of glucose homeostasis. However, the exact mechanisms by which the amino acid affects blood glucose levels are still unknown [17].

In this study, we investigate the effect of taurine on glucose intolerance, lipid profile, Hcy level, TAC, PON activity, and NOx concentration in HFD-induced IR. To the best of our knowledge, there was no previous focus on the effect of taurine supplementation on HHcy in insulin resistant rat model.

## Materials and methods

### Chemicals

Taurine (Oxford Chemical Company, India). Glucose and Zinc Sulphate (El Nasr Co, Cairo, Egypt). Phenyl acetate, Tris HCl buffer, sodium nitrite, vanadium (III) chloride, sulfanilamide and N-(1-naphthyl) ethylendiamine dihydrochloride (Sigma Chemical Company, USA). Calcium chloride (Fluka Chemical Company, USA).

### Diet

The control diet for the rats contained 60% starch, 20.7% casein, 0.3% methionine, 5% fat, 7.9% cellulose, 5% minerals and 1% vitamins mix. The fructose diet contained 60% fructose instead of starch (Harlan-Teklad, TD. 89247, Madison, WI, USA), while the remaining composition was the same.

### Animals

Thirty two male *Wistar *rats were used for the present study after being procured from the animal house of El-Nile Company for Pharmaceutical Products (Cairo, Egypt). The animals were acclimatized for two weeks in the animal house of Misr International University before dietary manipulation. Two rats were housed per wire floored cage in an air-conditioned room (22 ± 2°C) with 12 h light/dark cycle and had free access to standard laboratory chow diet (El Nasr Co, Cairo, Egypt), and water *ad libitum*. The protocol of the current study was approved by the Department of Biochemistry Council, Faculty of Pharmacy, Ain Shams University, which has an ethical authority.

### Experimental design

Animals weighed 170-190 g at the time of dietary manipulation. They were randomly assigned into four groups of eight each, as given below:

i. Control group (C): normal control rats, received control diet.

ii. Taurine group (C + T): taurine-treated normal rats, received taurine (300 mg/kg/day) via i.p. route [18], and control diet.

iii. Fructose-fed group (F): fructose-fed rats, received HFD.

iv. Fructose-fed + taurine group (F + T): taurine-treated fructose-fed rats, received taurine (300 mg/kg/day) via i.p. route, and HFD.

The animals were maintained in their respective groups for 35 days. OGTT were carried out and animals' body weights were measured at different intervals during the feeding period. Fasting serum glucose, insulin, lipid profile, TAC, PON, and NOx as well as plasma Hcy of all animals were measured at the last day (day 35) of the experiment.

### Oral glucose tolerance test (OGTT)

Twelve hours prior to days 0, 14, 28 and 35, rats were fasted and were subject to OGTT. For this, a glucose solution was introduced directly into the stomach of the conscious rats through a fine gastric catheter at a dose of 2 g/kg body weight [19]. Blood glucose levels were determined at 0 (before glucose administration), 30, 60, 90 and 120 min after glucose administration using an automated glucometer (One Touch-Horizon, Johnson & Johnson (Life Scan) blood glucose monitoring system, Almere, Netherlands).

### Sample collection

Blood samples were collected from retro-orbital plexus of the eye after 35 days from 12-h fasted rats into two different types of vacutainer tubes. The first contained EDTA as anticoagulant for the assay of Hcy, while the second was plain for serum preparation. The samples were centrifuged at 3000 rpm for 10 min at 4°C using Centurion centrifuge (K280R, UK). The plasma was then stored at -20°C for the assay of Hcy, while the serum was aliquoted and stored at -80°C for the assay of insulin, glucose, TGs, T-Chol, high density lipoprotein cholesterol (HDL-C), LDL-C, TAC, PON, and NOx. All assays were performed within two months after sample collection.

### Biochemical measurements

The concentration of serum glucose was measured by the enzymatic colorimetric GOD-POD procedure [20] using Diamond Diagnostics kit (Germany). Insulin was determined using an enzyme linked immunosorbent assay (ELISA) kit purchased from Linco research (USA) (Cat.# EZRMI-13K). The IR was estimated using the HOMA which is equal to: [fasting serum insulin (μU/ml) × fasting serum glucose (mmol/l)/22.5] [21].

Serum TGs were estimated by GPO-POD enzymatic method [22] using a Biocon kit (India). T-Chol and LDL-C concentrations were determined utilizing enzymatic colorimetric CHOD-PAP method [23,24] using Biocon kits (India). HDL-C was determined by the same method after the precipitation of very low density lipoprotein cholesterol (VLDL-C) and LDL-C [25], and finally, the atherogenic index (T-Chol/HDL-C) was calculated.

Plasma Hcy was assessed by a chemiluminescent technique using ARCHITECT *i*2000 immunoassay analyzer (Abbott Diagnostics, Germany). Serum TAC was determined by an enzymatic colorimetric method [26], using Biodiagnostic kit (Egypt). PON activity was determined spectrophotometrically using phenyl acetate as substrate [27]. Serum NOx levels were measured by Griess reaction [28].

### Statistical analysis

The results were expressed as means ± SEM. To determine the statistical significance of laboratory findings, multiple comparisons were achieved using ANOVA followed by Tukey test as post hoc test. The correlations between PON and TAC were tested by Pearson's coefficient (*r*). P-value ≤ 0.05 was considered statistically significant.

## Results

**Figure 1:**
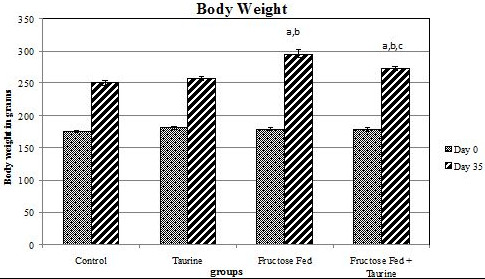
**Effect of HFD and taurine supplementation on weight gain at days 0 and 35 of feeding**. Each value is expressed in grams. (a) Significant difference from control group, at P ≤ 0.05. (b) Significant difference from taurine group, at P ≤ 0.05. (c) Significant difference from fructose-fed group, at P ≤ 0.05. Using one way ANOVA followed by Tukey test as post hoc test.

At day zero, there was no significant difference between the body weights of the four study groups. After 35 days of feeding, the HFD resulted in a significant increase in the body weight of group F by 18.1% when compared to group C, and by 15% when compared to group C + T. Taurine was able to attenuate this effect as group F + T was of a significantly less body weight than group F by 7.7% (Figure 1). Meanwhile, there was no significant difference in the water and food intakes among the four study groups.

**Figure 2:**
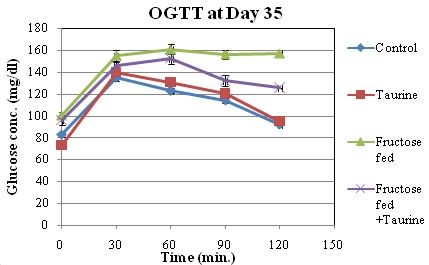
**Effect of HFD and taurine supplementation on OGTT at day 35 of the study**. Values are expressed as means ± SEM of 8 animals.

**Figure 3:**
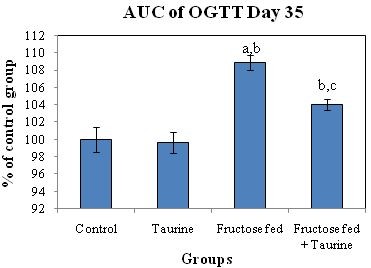
**Effect of HFD and taurine supplementation on AUC of OGTT at day 35 of the study**. Values are expressed as % of control group. (a) Significant difference from control group at day 35, at P ≤ 0.05. (b) Significant difference from taurine group at day 35, at P ≤ 0.05. (c) Significant difference from fructose-fed group at day 35, at P ≤ 0.05. Using one way ANOVA followed by Tukey test as post hoc test.

The analysis of the OGTT at the end of the feeding period and the comparison between area under the curve (AUC) of glycemia during 120 min from control and experimental groups showed that fructose-fed rats developed glucose intolerance (Table 1, Figure 2). The AUC of glucose during OGTT of group F was significantly elevated by 8.9% when compared to group C. The AUC of OGTT in group F + T was only elevated by 4% when compared to group C, which was statistically insignificant. The AUC of OGTT in group F + T was significantly lower than that of group F by 4.5%, showing improved glucose tolerance (Figure 3).

**Table 1:** Effect of HFD and taurine supplementation on the AUC of the OGTT at days 0, 14, 28, and 35:

Day	Group			
	
	Control	Taurine	Fructose-Fed	Fructose-Fed + Taurine
AUC day Zero	0.85 ± 0.011	0.84 ± 0.009	0.84 ± 0.009	0.83 ± 0.017

AUC day 14	0.83 ± 0.013	0.82 ± 0.008	0.90 ± 0.015^a,b^	0.87 ± 0.009^b^

AUC day 28	0.83 ± 0.014	0.81 ± 0.007	0.90 ± 0.014^a,b^	0.87 ± 0.017^b^

AUC day 35	0.84 ± 0.012	0.84 ± 0.010	0.92 ± 0.07^a,b^	0.88 ± 0.005^b,c^

Values are expressed as mean ± S.E.M. Number of rats per group n = 8.

HFD, high fructose diet; AUC, area under the curve; OGTT, oral glucose tolerance test.

(a) Significant difference from control group (P ≤ 0.05).

(b) Significant difference from taurine group (P ≤ 0.05).

(c) Significant difference from fructose-fed group (P ≤ 0.05).

**Figure 4:**
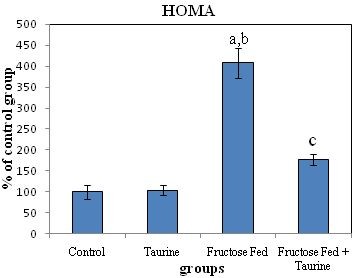
**Effect of fructose feeding and taurine supplementation on HOMA**. Each value is expressed as % of control group. (a) Significant difference from control group, at P ≤ 0.05. (b) Significant difference from taurine group, at P ≤ 0.05. (c) Significant difference from fructose-fed group, at P ≤ 0.05. Using one way ANOVA followed by Tukey test as post hoc test.

The HOMA results of group F were 4.1 fold greater than those of group C, and 3.9 fold greater than those of group C + T. Group F + T showed a significant increase when compared to groups C and C + T, but a significant decrease when compared to group F (Table 2, Figure 4).

**Table 2:** Effect of HFD and taurine supplementation on fasting serum glucose, serum insulin and HOMA:

Parameter	Group			
	
	Control	Taurine	Fructose-Fed	Fructose-Fed + Taurine
Glucose (mmol/l)	4.62 ± 0.28	4.09 ± 0.19	5.57 ± 0.25^a,b^	5.35 ± 0.23^b^

Insulin(μU/ml)	32.26 ± 4.76	38.24 ± 4.15	109.5 ± 7.37^a,b^	50.71 ± 4.45^c^

HOMA	6.68 ± 1.13	6.95 ± 0.79	27.24 ± 2.44^a,b^	11.9 ± 0.88^c^

Values are expressed as mean ± S.E.M. Number of rats per group n = 8.

HFD, high fructose diet; HOMA, homeostasis model assessment of insulin resistance.

(a) Significant difference from control group (P ≤ 0.05).

(b) Significant difference from taurine group (P ≤ 0.05).

(c) Significant difference from fructose-fed group (P ≤ 0.05).

There was no significant change in serum TGs between groups C and C + T. Meanwhile, there was a significant increase in that of group F relative to groups C and C + T, by 272% and 229.6%, respectively. The TGs level of group F + T showed a significant increase by 188.3% and 155.5% when compared to groups C and C + T, respectively, although, it was statistically lower than group F by 22.5% (Table 3).

**Table 3:** Effect of HFD and taurine supplementation on fasting serum lipids, TAC, PON, NOx and plasma Hcy:

Parameter	Group			
	
	Control	Taurine	Fructose-Fed	Fructose-Fed + Taurine
**TGs (mg/dl)**	70.47 ± 8.95	79.53 ± 7.94	262.16 ± 22.47^a,b^	203.17 ± 16.45^a,b,c^

**T-Chol (mg/dl)**	99.86 ± 2.21	93.32 ± 5.22	136.43 ± 1.92^a,b^	121.37 ± 2.59^a,b,c^

**LDL-C (mg/dl)**	29.30 ± 1.64	29.98 ± 3.22	57.27 ± 2.16^a,b^	45.04 ± 2.22^a,b,c^

**HDL-C (mg/dl)**	42.22 ± 1.20	41.35 ± 2.36	52.03 ± 1.40^a,b^	53.71 ± 1.79^a,b^

**Atherogenic index**	2.37 ± 0.03	2.27 ± 0.06	2.63 ± 0.06^a,b^	2.27 ± 0.07^c^

**Hcy (μmol/l)**	10.31 ± 0.65	11.02 ± 0.53	13.89 ± 0.48^a,b^	14.23 ± 0.62^a,b^

**TAC (mmol/L)**	3.05 ± 0.17	3.07 ± 0.11	2.44 ± 0.15^a,b^	3.11 ± 0.17^c^

**PON (U/ml)**	167.30 ± 9.08	160.92 ± 9.07	117.49 ±4.99^a,b^	148.51 ± 6.75^c^

**NOx (μM)**	21.70 ± 2	21.35 ± 1.66	41.97 ± 2.47^a,b^	26.82 ± 2.21^c^

Values are expressed as mean ± S.E.M. Number of animal per group n = 8.

HFD, high fructose diet; TGs, triglycerides; T-Chol, total cholesterol; LDL-C, low density lipoprotein cholesterol; HDL-C, high density lipoprotein cholesterol; TAC, total antioxidant capacity: PON, paraoxonase activity: NOx, nitric oxide metabolites: Hcy, homocysteine.

(a) Significant different from control group (P ≤ 0.05).

(b) Significant different from taurine group (P ≤ 0.05).

(c) Significant different from fructose-fed group (P ≤ 0.05).

There was no significant difference in serum T-Chol, LDL-C, and HDL-C between groups C and C + T, while there was a significant rise in all of them in group F relative to groups C and C + T. The atherogenic index of group F was significantly higher than those of groups C and C + T by 11% and 15.9%, respectively. Group F + T showed significantly lower levels of T-Chol and LDL-C when compared to group F, but they were still significantly higher than groups C and C + T. On the other hand, serum HDL-C concentrations of groups F and F + T were not statistically different from each other, but both were statistically higher than groups C and C + T. The atherogenic index of group F was statistically higher than those of groups C and C + T, while that of group F + T was statistically lower than that of group F but not statistically different from those of groups C and C + T (Table 3).

Plasma Hcy of groups F and F + T were not statistically different from each other but they showed a significant increase relative to groups C and C + T (Table 3).

**Figure 5:**
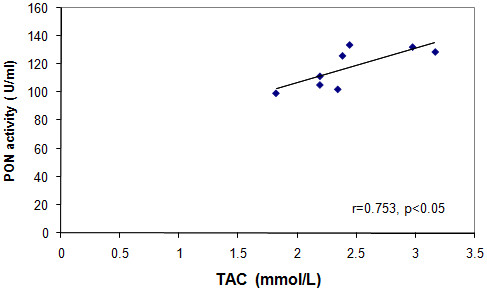
**Correlation between serum PON versus serum TAC in group F**.

**Figure 6:**
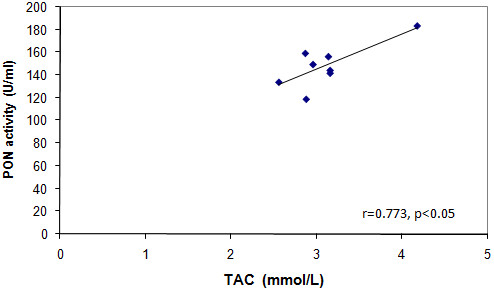
**Correlation between serum PON versus serum TAC in group F + T**.

Serum TAC and PON of group F were significantly lower than those of groups C and C + T, meanwhile taurine was able to abolish this effect in group F + T. Serum TAC positively correlated with serum PON in groups F (Figure 5) and F + T (Figure 6) at p < 0.05 (r = 0.753 and 0.773, respectively). Serum NOx of group F showed a significant increase when compared to groups C and C + T, an effect that was also abolished in the F + T group (Table 3).

## Discussion

The MS is a cluster of cardiovascular risk factors which include obesity, central obesity, atherogenic dyslipidemia and IR [29]. Apart from its association with CVD and diabetes mellitus, it is a common soil for numerous other clinical disorders [30].

Our work is comparable to that of Kannappan and Aduradha [31], who were able to show that the HFD was able to impair insulin sensitivity and glucose tolerance. This was revealed by the significant elevation in AUC of the OGTT of group F. Such a finding was confirmed by the observed hyperglycemia, hyperinsulinemia and elevated HOMA of the same group, and is matched with those of Yadav et al. [32].

Increases in the fructose load to the liver, could elicit rapid responses that ultimately influence hepatic gene expression, glucose disposal, and insulin action. This may be attributed to the fact that fructose metabolism bypasses the regulatory step catalyzed by phosphofructokinase-1. Thus, fructose continuously enters the glycolytic pathway resulting in hyperglycemia [33]. The extra glucose released into the blood stimulates more insulin secretion, leading to reduced insulin sensitivity [34].

Visceral adiposity is known to be increased by HFD [35]. It is associated with IR as a result of the direct delivery of portal blood flow from visceral fat to the liver releasing FFAs [36]. The greater lipolytic capacity of visceral than peripheral adipocytes releases more FFAs to the portal circulation. Furthermore, when visceral adipocytes enlarge, they become more insulin resistant than smaller adipocytes [37]. Increased amounts of FFAs directly affect insulin signaling, diminish glucose uptake in muscle, and induce gluconeogenesis in the liver [38].

Although taurine was unable to improve the fasting hyperglycemia, it was able to attenuate the elevated AUC of the OGTT, as well as the observed hyperinsulinemia, and it greatly improved the elevated HOMA. It was speculated that in this diet-induced model, activation of serine kinases coupled with inhibition of tyrosine phosphorylation of the insulin receptor could result in IR. Changes in redox balance can activate certain stress-induced serine kinases which can in turn decrease the extent of tyrosine phosphorylation, and is consistent with the attenuation of insulin action [39]. It was previously described that taurine modulates the insulin signal transduction pathways by inhibiting the cellular protein tyrosine phosphatase activity that negatively regulates insulin signaling. Thus, taurine has the potential ability to prolong as well as increase insulin signaling. It is also possible that taurine being an antioxidant, would make the cells less susceptible to the consequence of stress-induced activation of serine kinases [40].

The consumption of the HFD resulted in hypertriglyceridemia, hypercholesterolemia, and increased levels of both LDL-C and HDL-C. Fasting hypertriglyceridemia in IR has largely been attributed to apoB-100 containing TGs rich very low density lipoprotein (VLDL) overproduction and secretion by the liver, with a lesser contribution to the impaired VLDL removal [41]. Fructose consumption can promote hepatic lipogenesis because it provides unregulated amounts of lipogenic substrates acetyl-CoA and glycerol-3-phosphate [32]. Fructose can also activate sterol regulatory element binding protein-1c (SREBP-1c) independently of insulin, which then activates genes involved in *de-novo *lipogenesis [42]. SREBP-1c over-expression was also reported to inhibit insulin receptor substrate-2 expression, which might contribute to a transitional switch from glycogen synthesis to lipogenesis [43]. High density lipoprotein (HDL) is the major cholesterol lipoprotein carrier in rats [3], thus, the elevation of serum HDL-C could merely be a reflection to the observed increased serum T-Chol.

Taurine can upregulate 7-α-hydroxylase, the rate-limiting enzyme in bile acids production [44], and was shown to increase its mRNA levels [45]. Taurine may also decrease cholesterol levels through upregulation of hepatic LDL receptor and/or through improving the binding of LDL to them. Thus, it increases the LDL turnover in blood [46]. The ability of taurine to decrease the T-Chol level could be the main contributor to the reduced atherogenic index of group F + T.

The HHcy observed in the fructose-fed model of IR may be attributed to the reduction in the specific activity of two key enzymes of Hcy metabolism, namely, methyltetrahydrofolate reductase and cystathionine β synthase (CβS). Dicker-Brown et al. used cultured hepatocytes to show that chronic insulin addition was able to induce HHcy that was due to Hcy being transformed to either methionine or cysteine at a reduced rate [9].

Hyperhomocysteinemia could also be explained in light of the observed hypertriglyceridemia which might specifically promote lipid deposition in visceral adipose tissue as commonly associated with IR [36]. N-nicotinamide methyltransferase (NNMT) is a major methyltransferase expressed in high amounts in human adipose tissue [47]. It converts nicotinamide into N-methyl nicotinamide at the expense of S-adenosyl methionine as methyl-donating cofactor. The generated S-adenosyl homocysteine could further be converted to Hcy [35]. Thus, the observed HHcy in the fructose-fed rat model of the current study could be attributed to the increased visceral adiposity accompanying overconsumption of fructose.

The high levels of Hcy could be metabolized into Hcy thiolactone, a physiological substrate of PON protein. Hcy thiolactone can cause HDL homocysteinylation [48] and consequently decreases its PON activity as revealed by the results of the current work. Under conditions of high oxidative stress, PON may be inactivated by S-glutathionylation, a redox regulatory mechanism characterized by the formation of a mixed disulfide between a protein thiol (i.e. cysteine-284 of PON enzyme) and oxidized glutathione [49]. The lower PON activity observed in group F may also be due to the increased T-Chol that increases the susceptibility of LDL to oxidation. This process inactivates PON in an interaction between the lipid peroxides and the sulfhydryl groups of the enzyme as previously shown by Bajnok et al. [50]. Systemic oxidative stress is associated with IR, which manifests as decreased TAC. This is possibly due to increased oxidative stress on one hand and decreased activities of different antioxidative enzymes on the other. Thus, the significant positive correlations revealed between TAC and PON activities of groups F and F + T are convenient.

The significant reduction of TAC in group F could be attributed to the fact that the high fructose delivery to the liver may generate stress-activating molecules, such as methylglyoxal and/or d-glyceraldehyde. These molecules can serve as substrates for AGEs [51]. AGEs could activate NADPH oxidase in endothelial cells. Activation of NADPH oxidase could also occur cause endothelial cells lack CβS and Betaine:homocysteine methyltransferase. Thus, Hcy depends on the methionine synthase pathway for its elimination. HHcy may thus cause a deficiency of folic acid with subsequent deficiency of tetrahydrobiopetrin, and consequently, uncoupling of the endothelial NOS reaction producing superoxide anion (O _2_•^-^) as well as ONOO^- ^rather than NO [52]. Hcy was also shown to reduce the expression of glutathione peroxidase and the secretion and expression of extracellular superoxide dismutase [53,54]. Thus, in addition to directly producing ROS, Hcy also reduces the O _2_•^- ^anion scavenging capacity leading to further elevation of oxidative stress.

Hyperhomocysteinemia could also promote ROS production by increasing inducible NOS expression which subsequently increases nitrotyrosine formation [55]. This fact could explain the significant elevation of NOx observed in group F. ROS could also reduce NO bioavailability by inactivating it to ONOO^-^. In this respect, the elevation of serum NOx might indeed reflect the impaired NO bioavailability since ONOO^-^, as well as NO, are metabolized into nitrites and nitrates [56]. Recently, it has been shown that ONOO^-^, in the absence of known nitrosative stress-protecting enzymes, could be degraded by catalase enzyme into nitrate (70%) and nitrite (30%) [57]. A finding that may aid in rationalizing the elevated NOx concentration in this fructose-fed model.

Taurine is synthesized from cysteine, the precursor of glutathione (GSH). Hence, taurine supplementation may spare cysteine, thus increasing tissue levels of GSH, restoring TAC as well as PON activity back to normal [58]. In the present study, taurine supplementation was also able to improve the elevated NOx which may have been achieved through lowering inducible NOS gene expression as previously reported by Hsu et al. [59], or through scavenging O _2_•^- ^and NO, the precursors of ONOO^- ^under conditions of elevated oxidative stress [60]. These results are matched with those of Yalçınkaya et al. [61], who reported that taurine was not able to improve the diet-induced HHcy, although it was able to improve HHcy-induced ROS production. Thus, further studies are required to define or not whether different dosages and/or durations of taurine supplementation will be able to improve the observed HHcy in this model of IR.

## Conclusion

Our study revealed that rats fed on a HFD develop IR as manifested by hyperglycemia, hyperinsulinemia, and elevated HOMA. They showed metabolic dyslipidemia as well as HHcy. The HFD also resulted in decreased TAC, decreased PON activity, and increased NOx production. Our results demonstrate that taurine supplementation is able to improve the glucose intolerance, the hyperinsulinemia and to guard against the drastic increase in HOMA. Taurine is also able to improve dyslipidemia, and to abolish the effect of the HFD on TAC, PON activity, and NOx concentration. Meanwhile, it was not able to attenuate the observed HHcy associated with the development of IR in the current dose and duration.

## Competing interests

The authors declare that they have no competing interests.

## Authors' contributions

HOM put the idea of research and developed the study protocol, supervised samples and results interpretation and correlation assessments, and contributed to the revising of the manuscript. EE participated in protocol writing, as well as experimental work, and helped in statistical analysis. LNH supervised samples' collection, preparation and analysis, provided superb scientific guidance regarding interpretation and presentation of results, and helped to draft the manuscript. HMM carried the designed protocol, performed the statistical analysis and contributed to the writing of the manuscript. All authors read and approved the final manuscript.
